# Genomic sequence of temperate phage Smp131 of *Stenotrophomonas maltophilia* that has similar prophages in xanthomonads

**DOI:** 10.1186/1471-2180-14-17

**Published:** 2014-01-28

**Authors:** Chia-Ni Lee, Tsai-Tien Tseng, Hsiao-Chuan Chang, Juey-Wen Lin, Shu-Fen Weng

**Affiliations:** 1 Institute of Molecular Biology, National Chung Hsing University, Taichung 402, Taiwan; 2 Department of Biology and Chemistry, Southern Polytechnic State University, Marietta, GA 30060, USA; 3 Institute of Biochemistry, National Chung Hsing University, Taichung 402, Taiwan

**Keywords:** Genomic sequence, Integration, Prophage, *Stenotrophomonas*, Temperate phage, *Xanthomonas*

## Abstract

**Background:**

*Stenotrophomonas maltophilia* is a ubiquitous Gram-negative bacterium previously named as *Xanthomonas maltophilia*. This organism is an important nosocomial pathogen associated with infections in immunocompromised patients. Clinical isolates of *S. maltophilia* are mostly resistant to multiple antibiotics and treatment of its infections is becoming problematic. Several virulent bacteriophages, but not temperate phage, of *S. maltophilia* have been characterized.

**Results:**

In this study, a temperate myophage of *S. maltophilia* (Smp131) was isolated and characterized. Sequence analysis showed that its genome is 33,525-bp long with 47 open reading frames (ORFs). Its similarity to P2-like phages and prophages in *S. maltophilia* and several *Xanthomonas* pathovars includes genomic organization, arrangement of several operons, and possession of a slippery sequence T_7_G for translational frameshifting in tail assembly genes. Smp131 encodes a tyrosine family integrase that shares low degrees of similarity with those of other phages and a lysin belonging to family 19 chitinase that is observed in plants and some bacteria, although not in phages. tRNA are the preferred sites for host integration of Smp131 and the related phages: tRNA-Thr for Smp131 and prophage of *S. maltophilia* K279a; tRNA-Lys for prophages of *X. campestris* pv. campestris ATCC33913, *X. oryzae* pv. oryzae strains MAFF311018, and KACC10331; and tRNA-Asn for prophage of *X. oryzae* pv. oryzae PXO99A and remnant of *X. axonopodis* pv. citri 306. Regions flanking the prophages are varied highly in nucleotide sequence and rich in transposase genes, suggesting that frequent insertion/excision had occurred.

**Conclusions:**

Prevalence of closely related prophages in *Stenotrophomonas* and *Xanthomonads* may have contributed to the diversity of these closely related species owing to possible horizontal gene transfer mediated by the phages.

## Background

*Stenotrophomonas maltophilia*, previously named as *Pseudomonas maltophilia* and then *Xanthomonas maltophilia*[1], is an aerobic, Gram-negative, rod-shaped bacterium common in different environments. *S. maltophilia* can cause various types of nosocomial infections, resulting in high morbidity and mortality in severely immunocompromised and debilitated patients [2,3]. This organism is increasingly prevalent in hospitals worldwide; in Taiwan, it is ranked one of the highest occurring nosocomial infections [4]. In addition, isolates obtained from hospitalized patients show significant genetic diversity, suggesting that they can be derived from various sources [5]. Recently, treatment of *S. maltophilia* infections has become more difficult because of the high prevalence of multiple resistance to antibiotics of this organism [6].

Phage therapy has attracted significant attention for its effectiveness in treating bacterial infections [7]. Some *S. maltophilia* phages have been reported including i) two lytic phages (phiSMA5 and Smp14) from our laboratory that resemble members of *Myoviridae* in morphology with a genome of approximately 250 and 160 kb, respectively [4,8], ii) a T7-like phage lytic to pan-resistant *S. maltophilia* and a phage that has large burst size and unique plaque polymorphism, with their genomes being sequenced [9,10], iii) a phage remnant in *S. maltophilia* strain P28 that is capable of producing a novel phage tail-like bacteriocin, designated as maltocin P28 [11], iv) detection of a phage genome carrying a zonula occludens like toxin gene [12], and v) three filamentous phages [13,14]. In addition, we have described a novel lysozyme encoded by a *Xanthomonas oryzae* phage, phiXo411, that is active against both *Xanthomonas* and *Stenotrophomonas*[15]. Although the lytic phages, the lysozyme and the maltocin P28 are potentially useful in treating *S. maltophilia* infection, feasible testing has yet been reported.

In spite of the above mentioned efforts in phage study, no temperate phage of *S. maltophilia* has been reported. In this study, we isolated a temperate phage of *S. maltophilia* and designated as Smp131. Since acquisition of external DNA by horizontal gene transfer and gene loss are major driving-forces of bacterial genome evolution and integration and excision of temperate bacteriophages contribute actively to such evolution [16], we deemed it worthy to study this phage. The phage genome was sequenced and sequence analysis revealed that Smp131 is similar to phage P2 and shares high degrees of identity with prophages of *Stenotrophomonas* and xanthomonads.

## Results and discussion

### Phage Smp131 is a temperate myophage infecting *S. maltophilia*

In this study, temperate phages were detected by spotting culture supernatants from 86 clinical isolates of *S. maltophilia* onto lawns formed separately by all other isolates. The culture supernatant from *S. maltophilia* strain T13 was observed to cause clearing zones on 3 of the samples (ATCC 13637, BCRC 11901, and T16). Following 3 rounds of single plaque isolation, Smp131 was obtained and used for further study. Less turbid plaques were formed on lawns of strain T16; therefore, this strain was used as the host for phage propagation and indicator host in titering the phage.

**Figure 1:**
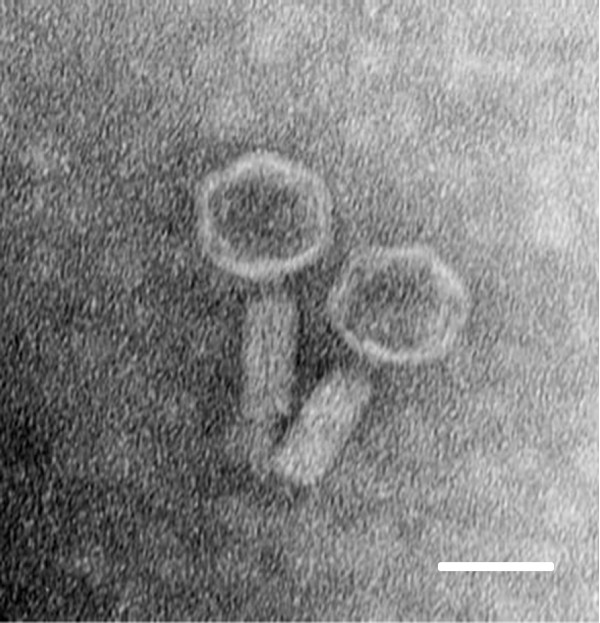
**Transmission electron micrograph of Smp131.** Samples were stained with 2% uranyl acetate. Scale bar represents 50 nm.

Cultures of *S. maltophilia* T13 released from 1 × 10^4^ to 1 × 10^6^ PFU/ml of Smp131 and treatment by adding mitomycin C (1 μg/ml) into the cultures produced titers of approximately 7 × 10^8^ PFU/ml. Electron microscopy showed that Smp131 has an icosahedral head approximately 60 nm in diameter and a contractile tail 100–120 nm in length and 20–30 nm in width (Figure 1), resembling members of *Myoviridae* phages.

In SDS-polyacrylamide gel (10%) electrophoresis, phage particles purified by CsCl ultracentrifugation displayed more than 15 distinct protein bands, with molecular masses ranging from 16 to 120 kDa, upon staining the gel with Coomassie brilliant blue. Four bands, with molecular masses of 44, 39.5, 38, and 21 kDa, were more abundant than the others. The 38-kDa protein was the most abundant and is likely the major capsid protein.

Host range testing showed that only the three *S. maltophilia* strains, ATCC 13637, BCRC 11901, and T16, were sensitive to Smp131 as indicated by the formation of single plaques. Several reasons are possible for the phage resistance, including immunity, impaired adsorption and block at later stages during phage infection, and further study is needed to test these possibilities. With such a narrow host range, Smp131 apparently has limited use in control of *S. maltophilia* infection. Spot tests and plaque assays were also tested on bacteria other than *S. maltophilia* strains, including *Escherichia coli* (n = 14, with n being the number of isolates)*, Serratia marcescens* (n =33)*, Enterobacter cloacae* (n = 12)*, Klebsiella pneumoniae* (n = 10)*, Proteus mirabilis* (n = 11)*, Pseudomonas aeruginosa* (n = 7), *Xanthomonas campestris* pv. campestris (n = 7), *X. axonopodis* pv. citri (n = 1), *X. axonopodis* pv. dieffenbachiae (n = 1), *X. axonopodis* pv. glycines (n = 1), *X. axonopodis* pv. phaseoli (n = 1), *X. axonapodis* pv. vesicatoria (n = 46), and *X. oryzae* pv. oryzae (n = 2). None of these bacteria were sensitive to Smp131, indicating that this phage has a narrow host range. This is different from phage P2 that can infect several enteric bacterial species [17].

### The circular Smp131 genome has a cohesive region conserved in P2-like phages

**Figure 2:**
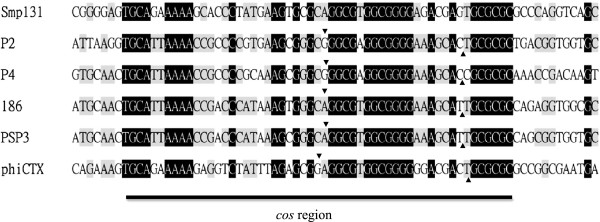
**Smp131 *cos *region deduced by analogy to those of P2-related phage.** The Smp131 sequence is aligned with the known *cos* regions of *Enterobacteria* phages P2 (GenBank:NC_001895) and P4 (GenBank:NC_001609), with arrowheads indicating *cos* cleavage sites [12]. Also aligned are corresponding regions from *Enterobacteria* phages 186 (GenBank:U32222) and PSP3 (GenBank:NC_005340), and *Pseudomonas* phage phiCTX (GenBank:NC_003278). CLUSTAL X1.83 was used for alignment. Letters with black and grey backgrounds are nucleotides identical in all and four or more sequences, respectively.

Restriction endonucleases AvaI, EcoRI, EcoRV, HincII, KpnI, NcoI, NotI, PstI, PvuII, and SphI were tested and found to be capable of cutting the Smp131 genomic DNA into distinct fragments. Sequencing of the Smp131 genome showed 33,525 bp, and 47 ORFs were identified (Additional file 1: Table S1). Nucleotide sequence comparison revealed that Smp131 had a region similar to the 55-bp *cos* region conserved in P2 and the related phages required for phage packaging [18]; GC-rich 19-nt 5′-extruding cohesive ends (5′-GGCGTGGCGGGGAGACGAG-3′) similar to those of P2-related phages (5′-GGCGAGGCGGGGAAAGCAC-3′) were observed in the *cos* region of Smp131 (Figure 2) [19]. By analogy to the P2 case, the extruding regions were set as the ends of the Smp131 genome.

The circularity of the Smp131 genome was demonstrated as follows. As shown in Additional file 2: Figure S1A, when displayed in a circular form, the left- and right-hand 19-nt extruding ends of the Smp131 genome would be paired. The genome had 6 EcoRI and 12 EcoRV sites, which were numbered from E1 to E6 and V1 to V12, respectively. Based on this predicted map, we isolated and sequenced a 2.5 kb EcoRI fragment (Additional file 2: Figure S1A). Results showed that this fragment was 2501-bp long, identical in nucleotide sequence to the E6-E1 region in the genome, and indeed contained the 19-bp *cos* site. To confirm circularity of the genome, fragment V12-E6 was used as the probe for Southern hybridization to probe a 4.7-kb EcoRV fragment (V12-V1). As anticipated, a 4.7-kb fragment was detected in the hybridization (Additional file 2: Figure S1B). These results indicate that Smp131 has a circular genome.

### Smp131 is similar to prophages in *Stenotrophomonas* and *Xanthomonas*

**Figure 3:**
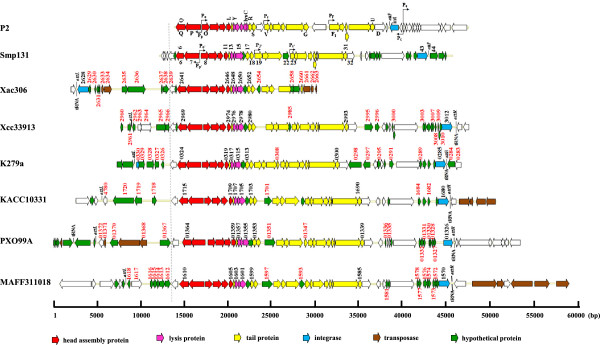
**Genome organization of phage Smp131, phage P2, and P2-like prophages in *Stenotrophomonas *and *Xanthomonas*.** Colored arrows indicate the directions and categories (denoted below) of the genes. The numbers or letters near the arrows indicate the names or locus_tags of the genes; red numbers indicate the homologues not found in Smp131. Bent arrows indicate the six promoters identified in phage P2 and those predicted for Smp131 by analogy. Abbreviations: P2, *Enterobacteria* phage P2; Xac306, prophage remnant in *X. axonopodis* pv. citri 306; Xcc33913, prophage in *X. campestris* pv. campestris ATCC33913; K279a, prophage in *S. maltophilia* K279a; KACC10331, PXO99A and MAFF311018, prophages in *X. oryzae* pv. oryzae strains KACC10331, PXO99A, MAFF311018, respectively.

Sequence analysis shows that Smp131 shares similarity with several prophages in genome organization and encoded proteins. They included 1) a 27-kb prophage remnant in *X. axonopodis* pv. citri strain 306; 2) a prophage each in *X. campestris* pv. campestris strain ATCC33913 (37 kb) and *X. oryzae* pv. oryzae strains KACC10331 (40 kb), MAFF311018 (37 kb) and PXO99A (42 kb); and 3) a 35-kb prophage in *S. maltophilia* K279a (Figure 3, Additional file 3: Table S2). Additionally, most Smp131-encoded proteins were similar to those encoded by several P2-like temperate phages (see below).

Similarity between Smp131 and prophages in *Xanthomonas* and *Stenotrophomonas* can be summarized as follows (Additional file 3: Table S2). First, genomes of these prophages (defined as the regions flanked by *attL* and *attR*, see below) were slightly larger than that of Smp131 (Figure 3), suggesting that some insertions in these prophages (Figure 3, numbered in red) and deletions (in/del) from Smp131 had occurred during evolution. Most of these in/dels encode hypothetical proteins. It is apparent that those absent from Smp131 are nonessential genes. Second, some Smp131 genes (*orf01, 02*, *03*, *05*, *22*, *29*, *36*, *38*, *41*, *44*, *45*, and *46*) were absent from one or more of the other prophages (remnant in *X. axonopodis* pv. citri strain 306 lacked *orf 01*, *02*, *03*, *23*–*40*, and *orf 44–46*). Third, there were transposase genes associated with the *Xanthomonas* prophages and remnant (Figure 3): 1) two in the upstream region and three in the downstream flanking region of the remnant, 2) four in the downstream flanking region of *X. oryzae* pv. oryzae KACC10331 prophage, 3) one in the upstream flanking region and three in the upstream of *X. oryzae* pv. oryzae PXO99A prophage, and 4) five in the downstream flanking region of *X. oryzae* pv. oryzae MAFF311018 prophage. Fourth, identity in amino acid sequence between corresponding proteins of Smp131 and these prophages ranged between 30% and 94%, with the majority falling above 50% (Additional file 3: Table S2). However, because none of their encoded proteins had been characterized, sequence comparison with proteins of these prophages did not lead to the identification of Smp131 gene functions.

Among the prophage harboring strains of *Stenotrophomonas* and *Xanthomonas*, *X. campestris* pv. campestris ATCC33913 was the only strain available to us. Spot test showed that the culture supernatant from *X. campestris* pv. campestris ATCC33913 did not form lysis zones on lawns of *X. campestris* pv. campestris strains Xc11 and Xc17, indicating that this strain may not release phage particles.

### The majority of Smp131-encoded proteins are similar to those of P2-like phages

No homologues were identified for proteins encoded by *orf1*, *orf2*, and *orf3* in the database, whereas *orf4* and *orf5* encoded a site-specific DNA methyltransferase and a hypothetical protein, respectively. Cluster *orf06* to *orf11* encoding capsid and packaging proteins was organized in the same order as P2 genes *QPONML*; *orf12* was similar to P2 gene *X*, annotated as tail protein (Additional file 1: Table S1, Figure 3).

**Figure 4:**
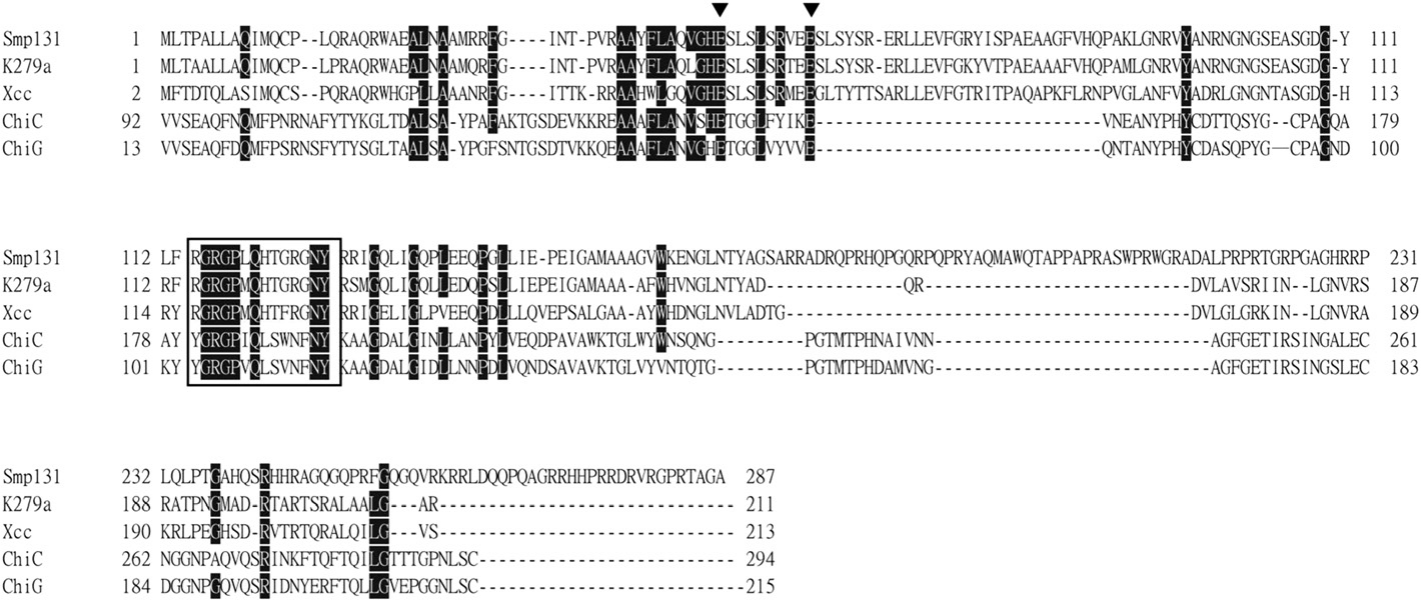
**Alignment of predicted Smp131 lysin with family 19 chitinases that have determined catalytic domains.** Identical residues are highlighted, with the conserved glutamate residues involved in catalysis indicated by downward arrowheads. The conserved sequence motif, [FHY]-G-R-G-[AP]-X-Q-[IL]-[ST]-[FHYW]-[HN]-[FY]-NY, that forms the substrate binding region is boxed. Abbreviations: Smp131, lysin encoded by *orf15* of Smp131; K279a, lysin encoded by prophage in *S. maltophilia* K279a (GenBank:YP_001970233); Xcc, lysin encoded by prophage in *X. campestris* pv. campestris ATCC33913 (GenBank:NP_638326); ChiC, chitinase C encoded by *Streptomyces griseus* (GenBank:YP_001824912); ChiG, chitinase G encoded by *S. coelicolor* (GenBank:BAA75648).

Proteins encoded by *orf13* and *orf14* possessed three transmembrane domains similar to Class I holins [20]. The product of *orf13* had a highly charged C terminus, which is characteristic of members of Class I, whereas ORF14 contained a slightly lower charged C terminus. *Orf15* was assigned as the endolysin gene. Rather than sharing similarity with phage lysozymes, the *orf15* product had a motif (aa 114–127) highly conserved in members of the GH19 chitinases family, [FHY]-G-R-G-[AP]-X-Q-[IL]-[ST]-[FHYW]-[HN]-[FY]-NY, that forms the substrate binding region [21] (Figure 4). Moreover, Glu50/Glu59 of ORF15 were similar to Glu68/Glu77 of *Streptomyces coelicolor* chitinase G experimentally identified as the active sites [22]. Family GH19 chitinases have long been identified in plants [23] and recently in bacteria [22,24-27], although not in phages; this Smp131 enzyme appears to be the first reported for phages.

Proteins encoded by *orf17* and *orf18* were homologous to *R* and *S* of P2, the tail completion proteins essential for stable head joining [28]. Proteins encoded by *orf19*, *orf20*, *orf23*, and *orf24* were homologous to that of the P2 *J, I, V*, and *W* (clustered with *H* and *G* as *VWJIHG*), respectively, whereas the position of *orf21* and *orf22* is similar to that of P2 *H* and *G*. Among the P2 genes, *VWJI* code for baseplate assembly proteins; H for putative tail fiber protein; and G for probable tail fiber assembly protein [29,30]. The difference in gene order suggests that rearrangement of these genes had occurred during evolution.

O*rf25* to *orf31*, except *orf29* that encoded a possible membrane protein, encoded tail proteins, whereas *orf32* encoded a late gene control protein. These genes corresponded to the P2 operon *F*_*I*_*F*_*II*_*EE’TUD* (Figure 3, Additional file 1: Table S1; [31]). In P2, E’ overlaps the start of gene *T*, lacks a potential ribosome binding site, and extends 37 nt back into *E* in the -1 reading frame. A run of 6 *T* residues (T_6_G slippery sequence) was located 20 nt upstream of the possible GUG start of *E’* and an extension of gene *E* following a -1 translational frameshift has been designated as *E + E’*[31]. The arrangement of *E* and *E’* genes within the tail gene cluster and their coupling through a translational frameshift is conserved among P2-related phages as well as in several other phages such as lambda although they share no similarity in amino acid sequence [31-33]. Near the 3′-end of *orf27*, there is a T_7_G similar to the conserved T_6_G slippery sequence [31], nt 288–295 relative to the *orf27* start codon. Thus, by analogy, a -1 translational frameshift may occur here during translation, thereby producing a protein product of *orf27.1* (Additional file 4: Figure S2A). Instead of the T_7_G, a predicted T_7_C slippery sequence was observed in the corresponding tail genes of prophages of *S. maltophilia* K279a, *X. campestris* pv. campestris 33913, *X. oryzae* pv. oryzae strains KACC10331, MAFF311018, and PXO99A (Additional file 4: Figure S2B). These findings indicate that this type of arrangement may be conserved in all P2-like phages.

The protein predicted for *orf33* was a phage-related protein similar to gp17 of phage BcepMu; *orf34* encoded a protein similar to that of P2 regulatory protein Ogr (see below); the products predicted for *orf35-46* were all hypothetical proteins, except that *orf39* and *orf43* encoded a DNA primase-like protein and a tyrosine family integrase, respectively. Tyrosine family integrases are responsible for DNA cleavage, strand exchange, and religation steps with a covalently bound phosphotyrosine intermediate [34]. As shown in Additional file 5: Figure S3, similarity search based on domain architecture [35] and sequence alignments showed that the predicted protein of *orf43* possessed 4 residues of the pentad conserved residues (R241, K264, H348 and H366) and the possible catalytic site Tyr375 (Additional file 5: Figure S3). However, no significant similarity in amino acid sequence was observed between the N-terminal region of Smp131 integrase and those of other integrases.

Varied degrees of identity were shared by Smp131 proteins with the analogous proteins from phages encompassing a wild host range (Figure 3, Additional file 6: Table S3). These homologues include 23 encoded by *Pseudomonas* phage phiCTX (27% to 73% identity), 22 by *Burkholderia* phage KL3 (34% to 62% identity), and 20 by *Enterobacteria* phage P2 (26% to 60% identity). The majority of these homologues cluster within the regions coding for capsid and packaging proteins (ORF06 to ORF11) as well as tail related proteins (ORF17 to ORF32, except ORF21 and ORF22). The cluster encoding lysis related proteins (ORF13 to ORF16) and the phage tail fiber protein (ORF21) shared lower degrees of identity, while ORF22 (hypothetical protein) shared no appreciable homology. The very recently reported P2-like phage remnant in *S. maltophilia* strain P28 possesses 23 *orf*s [11], nine of the deduced proteins share 31% to 53% identities with the Smp131 encoded proteins (Additional file 6: Table S3).

### Smp131 late genes may be regulated in a manner similar to that in P2

P2 has four late promoters, P_P_, P_O_, P_V_, and P_F_, possessing the consensus sequence TGT-N_12_-ACA and controlling *PQ*, *ONMLKRS*, *VWJIHG*, and *F*_*I*_*F*_*II*_*EE’TUD* operons, respectively [36,37]. Transcription of these operons depends on the Ogr protein, a zinc-finger containing transcriptional activator with a conserved cysteine motif, CX_2_CX_22_CX_4_C, where a zinc atom coordinates with four cysteine residues [38,39]. In Smp131, four putative late promoters were observed with sequences similar to TGT-N_12_-ACA, which were designated as P_P’_, P_O’_, P_J’_, and P_V’_ located at nt 4398–4381, 4381–4398, 10,964-10,981, and 14,928-14,946 in the genome, respectively (Figure 3). Operons presumably controlled by P_P’_ and P_O’_ were analogous to those by P2 P_P_ and P_O_, respectively, but those by P_J’_ and P_V’_ had some exchanged members due to gene rearrangement, that is, *VWJIHG* and *F*_*I*_*F*_*II*_*EE’TUD* in P2 versus *orf19*-*orf22* (homologous to *JIHG*) and *orf23-orf32* in Smp131 (Figure 3). Additionally, the protein encoded by Smp131 *orf34*, which had a relative position similar to that of the P2 Ogr gene, had a conserved CX_2_CX_22_CX_4_C motif, although overall similarity shared by the two proteins was low. Thus, similarity in genome organization, promoter sequence, and a regulatory protein suggests that Smp131 late genes are regulated in a manner similar to that in P2.

### tRNA genes are the preferred sites for integration of P2-like prophages of *Xanthomonas* and *Stenotrophomonas*

It is known that in *E. coli* i) P2 can integrate at over 10 different loci, with *locI* (*attB* site containing the core sequence, 5′-AAAAAATAAGCCCGTGTAAGGGAGATT-3′, which is identical to the *attP* sequence) being preferred over any other sites in *E. coli* C, ii) this site is occupied by a remnant of a P2 prophage in *E. coli* K-12 and P2 therefore will integrate into secondary sites, and iii) the P2 integrase accepts at least up to 37% mismatches within the core sequence [40]. Searching for a region similar to the P2 *attP* site in Smp131 genome revealed no such region. We then turned to identify putative *attR* and *attL* at the ends of prophage sequences from *Stenotrophomonas* and *Xanthomonas* and observed a 46-nucleotide perfect direct repeat at the extremities of the integrated prophage of *S. maltophilia* K279a, apparently representing *attL* and *attR* of the prophage (GenBank:NC_010943, Figure 3 and Additional file 7: Table S4). This 46-nucleotide sequence corresponded to the 3′-end of an intact tRNA-Thr gene. Nucleotide sequence comparison showed that a region identical to the *att* regions of the *S. maltophilia* K279a prophage was present in bp 30,738-30,783 (*orf43*/*orf44* intergenic region) of the Smp131 genome (Additional file 7: Table S4). This region, situated downstream of the integrase gene and similar in location to those in P2-like phages (phiCTX, GenBank:NC_003278; 186, GenBank:U32222), was thus predicted to be the *attP* site for Smp131 (Figure 3).

Based on the position of *attP*, we predicted that upon integration via *attP*, *orf44* and *orf43* would become flanked by *attL* and *attR*, respectively. In addition, an NaeI and a HincII restriction sites were located 644 bp and 667 bp relative to the *orf43* and *orf44* start codons, respectively, in the Smp131 genome (Additional file 8: Figure S4). Sequencing revealed that the amplicons were 1,092 bp and 704 bp containing *attL* and *attR*, respectively, which had a sequence identical to that of the Smp131 *attP*. To verify the *att*-flanking sequences, primers L3/L4 and R2/R3 were used to amplify the junctions of *attL* and *attR* regions, respectively (Additional file 8: Figure S4). Sequencing of these 2 replicons confirmed that our inverse PCR reactions had faithfully amplified the targeted regions. The result revealed that a segment of a possible defective integrase gene (480 bp) downstream of the *attL* was similar to that of *Burkholderia thailandensis* E264 (GenBank:YP_441483), whereas a 177-bp long host chromosomal region upstream of the *attR* was highly similar to the sequence adjacent to the tRNA-Thr of *S. maltophilia* strains (K279a and R551-3). These results suggest that upstream regions of tRNA-Thr are conserved in different strains of *S. maltophilia*, whereas the downstream regions are not. It was also noticed that upon integration, an intact tRNA-Thr that included the *attR* was regained, similar to the target site duplication observed by Rocco et al. [41].

In addition to *S. maltophilia* strain K279a (GenBank:NC_010943), the genome sequence has been determined for strain R551-3 (GenBank:NC_011071) [42,43]; they each had only one copy of tRNA-Thr located near one o’clock relative to the origin of chromosome replication (*ori*), as identified by containing DnaA boxes and genes involved in the initiation of bacterial chromosome replication [44]. Therefore, it is highly probable that this tRNA-Thr is the preferred site for Smp131 integration. Sequence analysis of junctions of integrated *Xanthomonas* prophage suggests that 1) prophages of *X. campestris* pv. campestris strain ATCC33913, and *X. oryzae* pv. oryzae strains MAFF311018 and KACC10331 integrated into a 45-bp region corresponding to 3′-end of a tRNA-Lys gene (GenBank:XCC3013, GenBank:XOO_r26, GenBank:XOO4676), 2) prophage of *X. oryzae* pv. oryzae PXO99A integrated into a 46-bp region corresponding to the 3′ end of a tRNA-Asn (GenBank:PXO_rna33), and 3) prophage remnant of *X. axonopodis* pv. citri 306 used the same sequence (GenBank:XAC2627) as that of *X. oryzae* pv. oryzae PXO99A prophage for integration, except that only *attL* was retained (Figure 3, and Additional file 7: Table S4). All identified *attB* sites for *Xanthomonas* are also located near one o’clock on the bacterial chromosomes.

Host integration of P2-like phages involves binding of integrase to the two arm-binding sites flanking the imperfect repeat, each having two direct repeats [45]. Careful examination of the Smp131 sequence revealed a pair of perfect direct repeats (5′-AATTTTACCGG-3′, bp 30635–30645 and bp 30647–30657) and an inverted repeat (5′-AAAAAGGCCAGCGCACCGCGCTGGCCTTTTT-3′, bp 30665–30695) in the upstream of *attP* (after the integrase gene, *orf43*), but no such sequences were found between *attP* and *orf44*. By analogy, it is possible that these repeats are involved in recognition by Smp131 integrase for host integration. However, lack of conserved repeats in the downstream suggests that the Smp131 integrase may be less demanding for sequence conservation in the downstream region for the function.

## Conclusions

This study is the first to isolate a temperate phage of *S. maltophilia*, Smp131. It is identified as a P2-like phage based on similarities to P2 in amino acid sequences of the encoded proteins, genomic organization, arrangement of several operons, and possession of a slippery sequence T_7_G for translational frameshifting in tail assembly genes. Smp131 is able to infect only *S. maltophilia*, different from phage P2 that can infect several enteric bacterial species. Several P2-like prophages in *S. maltophilia* and xanthomonads are also identified by bioinformatic analyses. In contrast to P2 that can integrate into several loci of the host chromosome, with certain loci being favoured and none of them being t-RNA gene, single t-RNA genes are found to be the locus for integration of these *Stenotrophomonas* and xanthomonads prophages. In addition, the regions flanking the prophages are rich in transposase-like genes, suggesting frequent exchange of genes during evolution. Existence of closely related prophages in *Stenotrophomonas* and xanthomonads is consistent with the close relatedness of these bacteria and the previous classification including *Stenotrophomonas* in genus *Xanthomonas*. Prevalence of the phages may have contributed to diversity of these closely related species owing to possible horizontal gene transfer mediated by the phages. With a narrow host range, the value to use Smp131 for controlling *S. maltophilia* infection is apparently limited.

## Methods

### Bacterial strains and growth conditions

Bacterial strains used in this study have been described previously [4]. *S. maltophilia* strains ATCC13637, BCRC 11901 and BCRC 15678 were used as reference strains [4]. Strain T16 was the host for propagation of phage Smp131 and as the indicator host in plaque assay. Luria-Bertani (LB) broth and LB agar plate were the general-purpose media [46] used to cultivate *S. maltophilia* (30°C), *Escherichia coli* (37°C), *Serratia marcescens* (37°C)*, Enterobacter cloacae* (37°C)*, Klebsiella pneumoniae* (37°C)*, Proteus mirabilis* (37°C)*, Pseudomonas aeruginosa* (37°C), and *Xanthomonas* strains (28°C).

### Spot test, isolation of bacteriophage and plaque assay

To detect the presence of phage in the culture supernatants and the phage sensitivity of a bacterium, spot tests were performed as described previously [4], except that LB broth and LB agar plates were used. The top agar containing the clearing zones was picked and soaked for 30 min in 100 μl of LB broth. Following appropriate dilution, the suspensions were plated for single plaque formation. Two more rounds of single-plaque isolation were performed to obtain the pure phage culture. To determine the phage titers, double-layered bioassays were performed on LB agar plates in which the top and bottom layers contained 0.75% and 1.5% agar, respectively. One-tenth of a milliliter each of a phage suspension after serial dilutions and cells of *S. maltophilia* strain from an overnight culture were mixed with 3 ml of molten soft agar and poured onto the bottom solidified agar (12 ml). Numbers of plaques were counted after the plates were incubated overnight. The same method was used to confirm phage susceptibility with the cells of different bacteria as the indicator hosts.

### Purification of phage particles

High-titer lysates of Smp131 (400 ml, approximately 1.0 × 10^10^ PFU/ml) were centrifuged (10,000 × g, 20 min at 4°C). The supernatants were passed through a membrane filter (0.45 μm pore size) and then centrifuged (15,000 × g at 4°C) for 2 hr. The phage pellets were suspended in 1.0 ml of the SM buffer (50 mM Tris–HCl, pH 7.5, containing 100 mM NaCl, 10 mM MgSO_4_, and 0.01% gelatin) and loaded on the block gradient of CsCl (1.2, 1.35, 1.45, 1.50, and 1.70 g/ml), followed by ultracentrifugation (28,000 rpm for 2 h at 4°C) with rotor TH641 (Sorvall OTD Combi) [15]. The phage particles concentrated into a zone were recovered and dialyzed against the SM buffer.

### DNA techniques

Phage particles purified following ultracentrifugation were treated with sodium dodecyl sulfate (SDS, 1%) and 20 U of proteinase K (Sigma P-2308) at 58°C for 1 h. An equal volume of phenol/chloroform (1:1) was then added to remove the proteinaceous materials. Phenol/chloroform extraction was repeated twice and the DNA was precipitated as described previously [47]. Restriction enzyme digestion of the phage DNA was performed in accordance with supplier instructions. DNA fragments were separated in 0.7% agarose gels in a TAE buffer (40 mM Tris acetate, pH, 8.0, containing 2 mM EDTA). Isolation of DNA fragments from agarose gel was performed using commercial kits (Qiagen). Standard protocols were followed for blotting DNA fragments onto the membrane (NEN catalog number NEF988), preparation of probes by labeling with [α-^32^P] dCTP (Du Pont. NEN), and Southern hybridization.

### DNA sequencing and bioinformatics

Processes for purification and shearing of phage DNA, cloning the DNA fragments into pBluescript II SK, and determination of the nucleotide sequence were performed as detailed in previous research [48]. Gaps were closed by primer walking with PCR-amplification on Smp131 genomic DNA as the template using primers designed according to available sequences. Programs used for DNA sequence analysis and similarity search based on domain architecture were selected according to previous research [49]. Possible ORFs were searched in 6 reading frames on both strands of the Smp131 genomic DNA, which used ATG or GTG as the start codon, consisted of longer than 50 amino acid residues, and had a putative ribosomal binding site in the upstream region.

The 33,525-bp DNA sequence determined in this study for phage Smp131 has been deposited in GenBank under accession number JQ809663.

### Cloning of the *attL* and *attR* regions flanking the Smp131 prophage

To clone the junction regions containing *attL* and *attR*, an inverse PCR-based strategy was employed. The chromosome prepared from *S. maltophilia* T13, the Smp131 lysogenic strain, was cleaved with NaeI and HincII separately and self-ligated to circularize the DNA molecules. Inverse PCR was performed using the circularized HincII and NaeI fragments as the templates with primer pairs L1/L2 (for amplification of the *attL*-containing region) and R1/R2 (for amplification of the *attR-*containing region), respectively. The amplicons obtained were sequenced for comparison.

### Separation of virion proteins by SDS-polyacrylamide gel electrophoresis

Following dialysis, phage particles (approximately 1 × 10^8^ PFU) purified by ultracentrifugation were boiled in a loading buffer for 3 min and separated in SDS-PAGE (10% polyacrylamide and 0.1% SDS). Protein bands were visualized by staining the gel with Coomassie brilliant blue (Bio-Rad) [47].

### Electron microscopy

Phage Smp131 was examined by electron microscopy of negatively stained preparations as described previously [4] using a JEM-1200 EX II transmission electron microscope (JEOL, Peabody, Mass) operated at 120 kV.

## Competing interests

The authors declare that they have no competing interests.

## Authors’ contributions

SFW designed the experiments. CNL and HCC carried out the wet lab. TTT and CNL performed bioinformatic analyses. JWL and TTT edited the manuscript. All authors read and approved the final manuscript.

## Supplementary Material

Additional file 1: Table S1 Assignment of Smp131 genes. Click here for file

Additional file 2: Figure S1 Strategy employed to test whether Smp131 has a circular form of genome. Lines: 1, restriction map deduced from the Smp131 sequence determined in this study; 2, fragments E1-3 (2.5 kb) and E5B1 (0.7 kb) used as probes for Southern hybridization; 3 and 4, 4.7-kb AvaI fragment (A1) and 4.7-kb EcoRV fragment (B5), respectively, that would hybridize to probes EI-3 and E5BI should the genome be circular. (B) Southern hybridization of AvaI and EcoRV digests from Smp131 genome using E1-3 and E5B1 separately as probes. Click here for file

Additional file 3: Table S2 Comparison of proteins deduced from prophages or remnant in *Xanthomonas* and *Stenotrophomonas*. Click here for file

Additional file 4: Figure S2 Predicted T7G translational frameshift sites in Smp131 and closely related prophages from *Xanthomoas* and *Stenotrophomonas*. **(A)** T7G (enclosed by a rectangle) and the surrounding regions including genes p27, p27.1 and p28 of Smp131. Stop codons are denoted by three dots after the amino acids. Predicted start codon ATG of p27.1 is underlined, whereas ribosomal binding site AGAGG for gene p28 is in gray background. **(B)** DNA sequence alignment of the regions surrounding T7G translational frameshift sites (enclosed in rectangles) from Smp131 and the related prophages from *X. campestris* pv. campestris 33913, *X. oryzae* pv. oryzae strains KACC10331, MAFF311018 and PXO99A. An asterisk indicates identical nucleotides in all phages. Click here for file

Additional file 5: Figure S3 Comparison of tyrosine integrase of Smp131 and its homologues. Identical residues found in more than 3 residues are highlighted. Active sites determined for XerD are indicated by downward arrowhead and the RKHRH pentad conserved residues are indicated above. The α-helix (empty rectangle) and β-sheet (empty arrow) structural motifs under the alignments are based on the crystal structure of *E. coli* XerD. Abbreviations: Smp131, integrase deduced from Smp131 *orf43*; P2, integrase of *Enterobacteria* phage P2 (GenBank:P36932); 186, integrase of *Enterobacteria* phage 186 (GenBank:P06723); XerD, site-specific recombinase of *E. coli* (GenBank:1A0P_A). Click here for file

Additional file 6: Table S3 Identities of amino acid sequence shared between the proteins deduced from Smp131 and those from bacteriophages. Click here for file

Additional file 7: Table S4 Positions and sequences of *att* sites and tRNA of Smp131 and prophages in *Xanthomonas* and *Stenotrophomonas.* Click here for file

Additional file 8: Figure S4 Strategy for cloning the host-prophage junctions from Smp131-lysogenized *S. maltophilia* T13. **(A)** Sketch depicting the circular Smp131 genome and genes near the predicted *attP* site. Arrows represent the genes and predicted *attP* site. **(B)** Sketch showing the host *S. maltophilia* T13 chromosome and its *attB* site. **(C)** Map showing relative positions of genes after Smp131 integration into host *S. maltophilia* T13. Primers used in PCR were: L1; 5′-TGAAAGGTGCCATGACCACACG-3′; L2, 5′-GCGTTGCCAAGGTCAGATCGG-3′; L3; 5′-CGCATCGCACTCTAGGAAGTGAAG-3′; L4, 5′-AACTGCCAGAACCTCTGCAGTG-3′; R1, 5′-CTCTTGTCCTCGCTGTCGGT-3′; R2, 5′-TGATAGCCCTATTTTCAAGGGC-3′; R3, 5′-AGGCCCAGCAGCGCA-3′; R4, 5′-TGCCTGCCGCCAGCT-3′. *S. maltophilia* T13 chromosome containing prophage Smp131 was digested with HincII and NaeI. The fragments were self-ligated and the circularized DNA was then used as the templates for inverse PCR. Amplicons obtained were sequenced for comparison. Click here for file
