# Supplementary material for: Genomic sequence of temperate phage Smp131 of Stenotrophomonas maltophilia that has similar prophages in xanthomonads
Source: BMC Microbiol. 2014 Jan 28;14:17. doi: 10.1186/1471-2180-14-17 (PMC3931495; doi:10.1186/1471-2180-14-17)
Supplement: Additional file 2: Figure S1 — Strategy employed to test whether Smp131 has a circular form of genome. Lines: 1, restriction map deduced from the Smp131 sequence determined in this study; 2, fragments E1-3 (2.5 kb) and E5B1 (0.7 kb) used as probes for Southern hybridization; 3 and 4, 4.7-kb AvaI fragment (A1) and 4.7-kb EcoRV fragment (B5), respectively, that would hybridize to probes EI-3 and E5BI should the genome be circular. (B) Southern hybridization of AvaI and EcoRV digests from Smp131 genome using E1-3 and E5B1 separately as probes. [file 1471-2180-14-17-S2.ppt]

## Slide 1
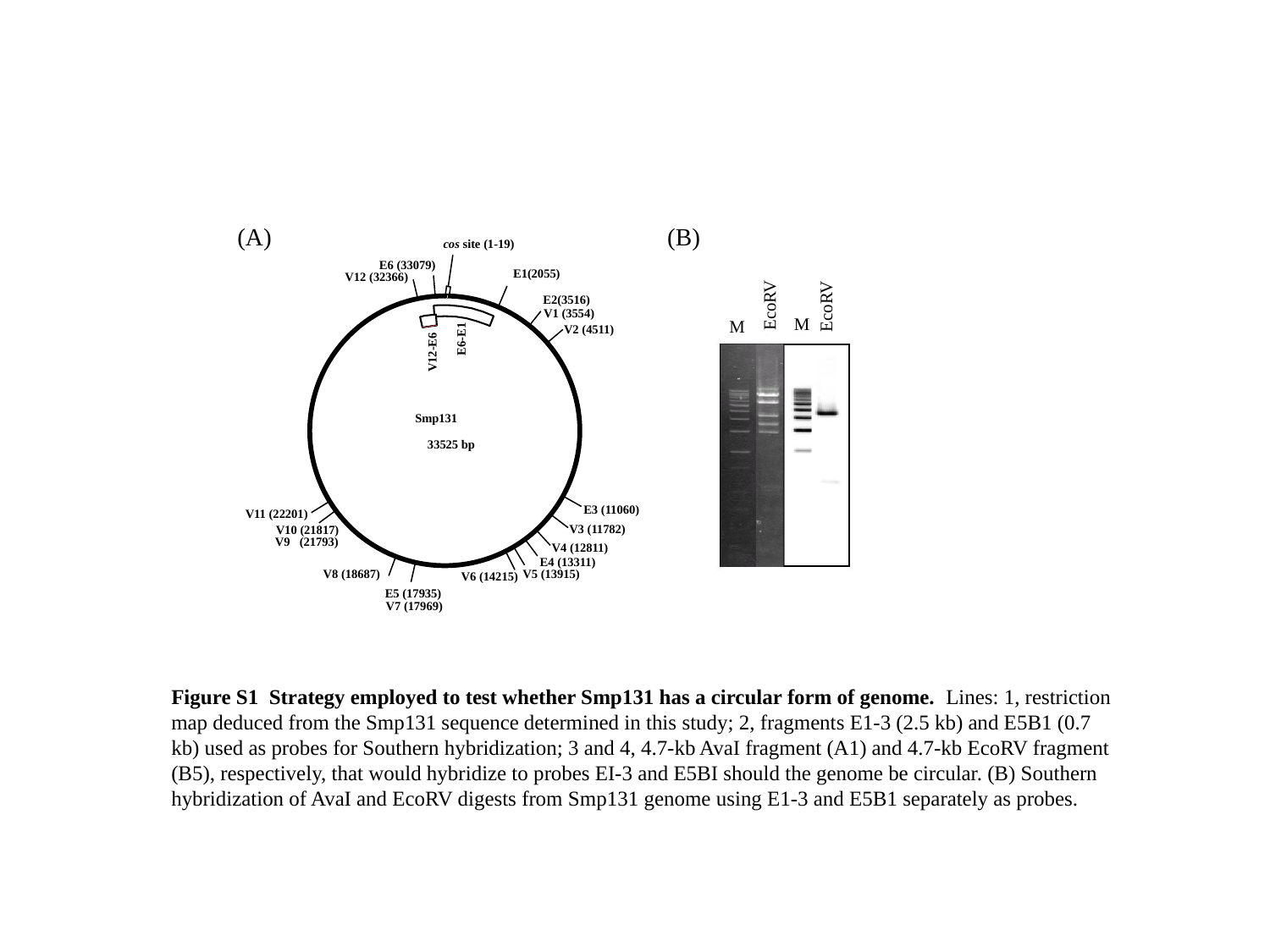

(A)
(B)
cos site (1-19)
E6 (33079)
E1(2055)
EcoRV
EcoRV
M
M
V12 (32366)
E2(3516)
V1 (3554)
V2 (4511)
E6-E1
V12-E6
Smp131
33525 bp
E3 (11060)
V11 (22201)
V3 (11782)
V10 (21817)
V9 (21793)
V4 (12811)
E4 (13311)
V8 (18687)
V5 (13915)
V6 (14215)
E5 (17935)
V7 (17969)
Figure S1 Strategy employed to test whether Smp131 has a circular form of genome. Lines: 1, restriction map deduced from the Smp131 sequence determined in this study; 2, fragments E1-3 (2.5 kb) and E5B1 (0.7 kb) used as probes for Southern hybridization; 3 and 4, 4.7-kb AvaI fragment (A1) and 4.7-kb EcoRV fragment (B5), respectively, that would hybridize to probes EI-3 and E5BI should the genome be circular. (B) Southern hybridization of AvaI and EcoRV digests from Smp131 genome using E1-3 and E5B1 separately as probes.
