# Supplementary material for: Genomic sequence of temperate phage Smp131 of Stenotrophomonas maltophilia that has similar prophages in xanthomonads
Source: BMC Microbiol. 2014 Jan 28;14:17. doi: 10.1186/1471-2180-14-17 (PMC3931495; doi:10.1186/1471-2180-14-17)
Supplement: Additional file 4: Figure S2 — Predicted T7G translational frameshift sites in Smp131 and closely related prophages from Xanthomoas and Stenotrophomonas. (A) T7G (enclosed by a rectangle) and the surrounding regions including genes p27, p27.1 and p28 of Smp131. Stop codons are denoted by three dots after the amino acids. Predicted start codon ATG of p27.1 is underlined, whereas ribosomal binding site AGAGG for gene p28 is in gray background. (B) DNA sequence alignment of the regions surrounding T7G translational frameshift sites (enclosed in rectangles) from Smp131 and the related prophages from X. campestris pv. campestris 33913, X. oryzae pv. oryzae strains KACC10331, MAFF311018 and PXO99A. An asterisk indicates identical nucleotides in all phages. [file 1471-2180-14-17-S4.ppt]

## Slide 1
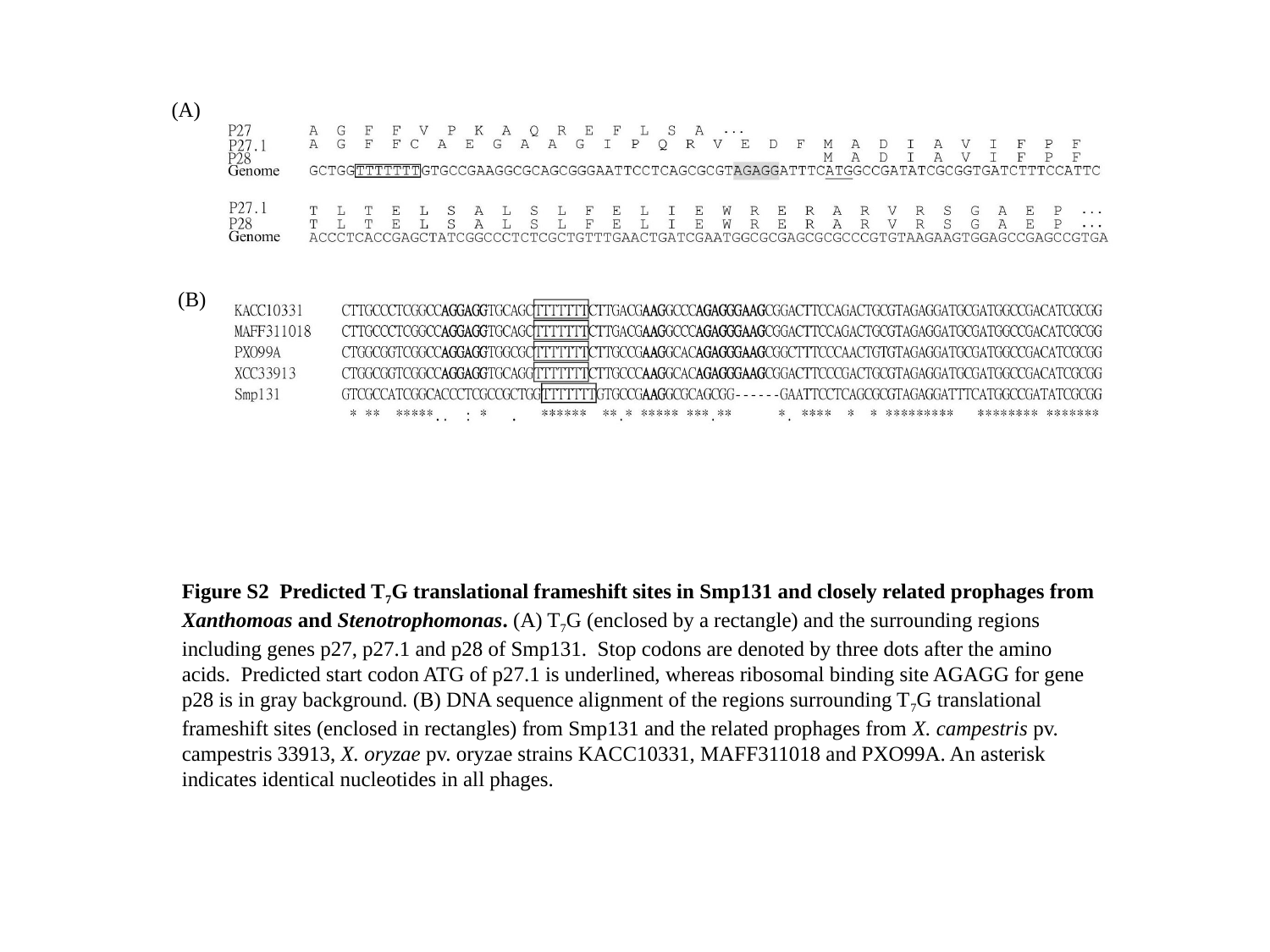

(A)
(B)
Figure S2 Predicted T7G translational frameshift sites in Smp131 and closely related prophages from Xanthomoas and Stenotrophomonas. (A) T7G (enclosed by a rectangle) and the surrounding regions including genes p27, p27.1 and p28 of Smp131. Stop codons are denoted by three dots after the amino acids. Predicted start codon ATG of p27.1 is underlined, whereas ribosomal binding site AGAGG for gene p28 is in gray background. (B) DNA sequence alignment of the regions surrounding T7G translational frameshift sites (enclosed in rectangles) from Smp131 and the related prophages from X. campestris pv. campestris 33913, X. oryzae pv. oryzae strains KACC10331, MAFF311018 and PXO99A. An asterisk indicates identical nucleotides in all phages.
