# Supplementary material for: Genomic sequence of temperate phage Smp131 of Stenotrophomonas maltophilia that has similar prophages in xanthomonads
Source: BMC Microbiol. 2014 Jan 28;14:17. doi: 10.1186/1471-2180-14-17 (PMC3931495; doi:10.1186/1471-2180-14-17)
Supplement: Additional file 5: Figure S3 — Comparison of tyrosine integrase of Smp131 and its homologues. Identical residues found in more than 3 residues are highlighted. Active sites determined for XerD are indicated by downward arrowhead and the RKHRH pentad conserved residues are indicated above. The α-helix (empty rectangle) and β-sheet (empty arrow) structural motifs under the alignments are based on the crystal structure of E. coli XerD. Abbreviations: Smp131, integrase deduced from Smp131 orf43; P2, integrase of Enterobacteria phage P2 (GenBank:P36932); 186, integrase of Enterobacteria phage 186 (GenBank:P06723); XerD, site-specific recombinase of E. coli (GenBank:1A0P_A). [file 1471-2180-14-17-S5.ppt]

## Slide 1
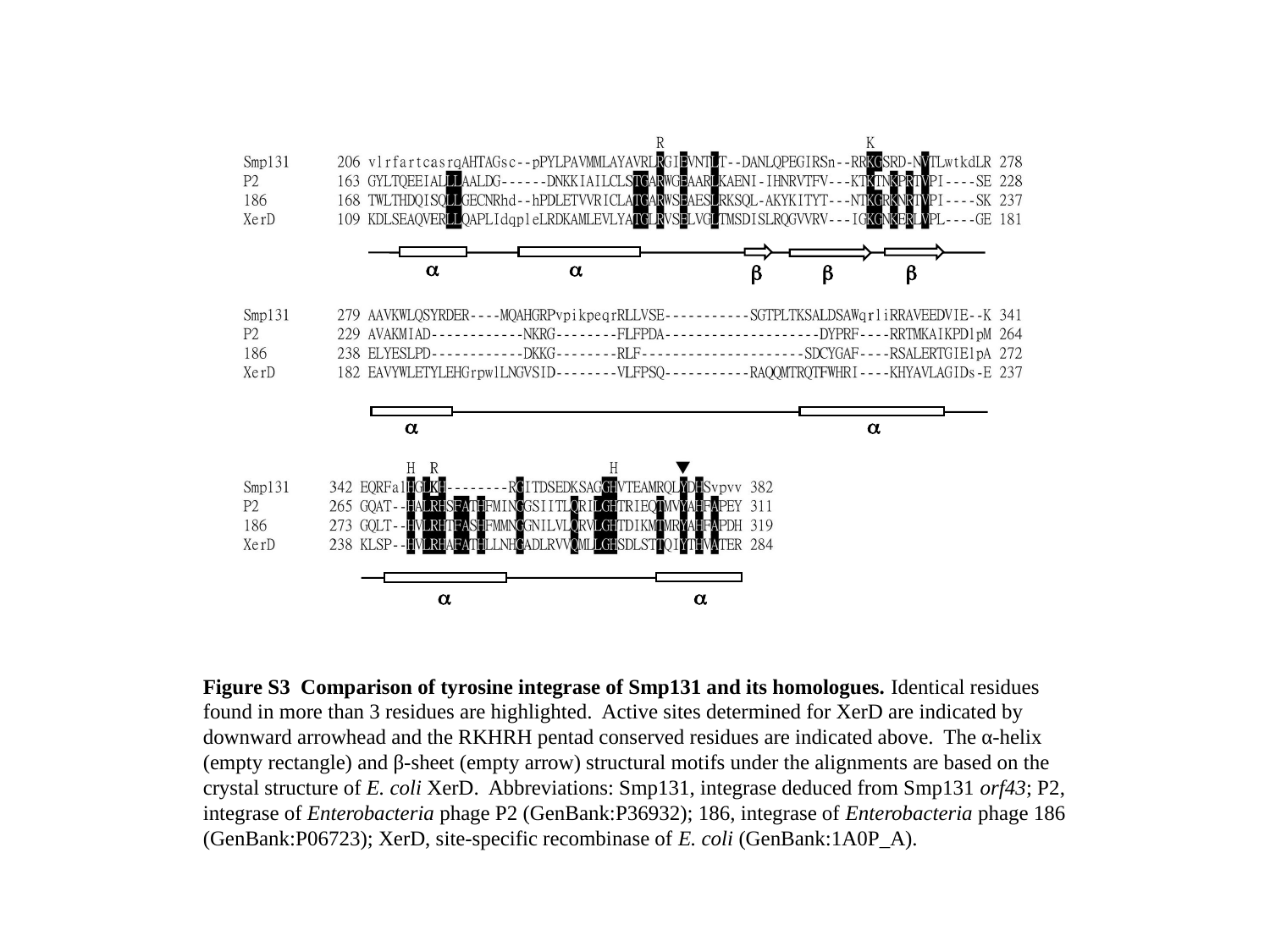

α
α
β
β
β
α
α
α
α
Figure S3 Comparison of tyrosine integrase of Smp131 and its homologues. Identical residues found in more than 3 residues are highlighted. Active sites determined for XerD are indicated by downward arrowhead and the RKHRH pentad conserved residues are indicated above. The α-helix (empty rectangle) and β-sheet (empty arrow) structural motifs under the alignments are based on the crystal structure of E. coli XerD. Abbreviations: Smp131, integrase deduced from Smp131 orf43; P2, integrase of Enterobacteria phage P2 (GenBank:P36932); 186, integrase of Enterobacteria phage 186 (GenBank:P06723); XerD, site-specific recombinase of E. coli (GenBank:1A0P_A).
