# Supplementary material for: Genomic sequence of temperate phage Smp131 of Stenotrophomonas maltophilia that has similar prophages in xanthomonads
Source: BMC Microbiol. 2014 Jan 28;14:17. doi: 10.1186/1471-2180-14-17 (PMC3931495; doi:10.1186/1471-2180-14-17)
Supplement: Additional file 8: Figure S4 — Strategy for cloning the host-prophage junctions from Smp131-lysogenized S. maltophilia T13. (A) Sketch depicting the circular Smp131 genome and genes near the predicted attP site. Arrows represent the genes and predicted attP site. (B) Sketch showing the host S. maltophilia T13 chromosome and its attB site. (C) Map showing relative positions of genes after Smp131 integration into host S. maltophilia T13. Primers used in PCR were: L1; 5′-TGAAAGGTGCCATGACCACACG-3′; L2, 5′-GCGTTGCCAAGGTCAGATCGG-3′; L3; 5′-CGCATCGCACTCTAGGAAGTGAAG-3′; L4, 5′-AACTGCCAGAACCTCTGCAGTG-3′; R1, 5′-CTCTTGTCCTCGCTGTCGGT-3′; R2, 5′-TGATAGCCCTATTTTCAAGGGC-3′; R3, 5′-AGGCCCAGCAGCGCA-3′; R4, 5′-TGCCTGCCGCCAGCT-3′. S. maltophilia T13 chromosome containing prophage Smp131 was digested with HincII and NaeI. The fragments were self-ligated and the circularized DNA was then used as the templates for inverse PCR. Amplicons obtained were sequenced for comparison. [file 1471-2180-14-17-S8.ppt]

## Slide 1
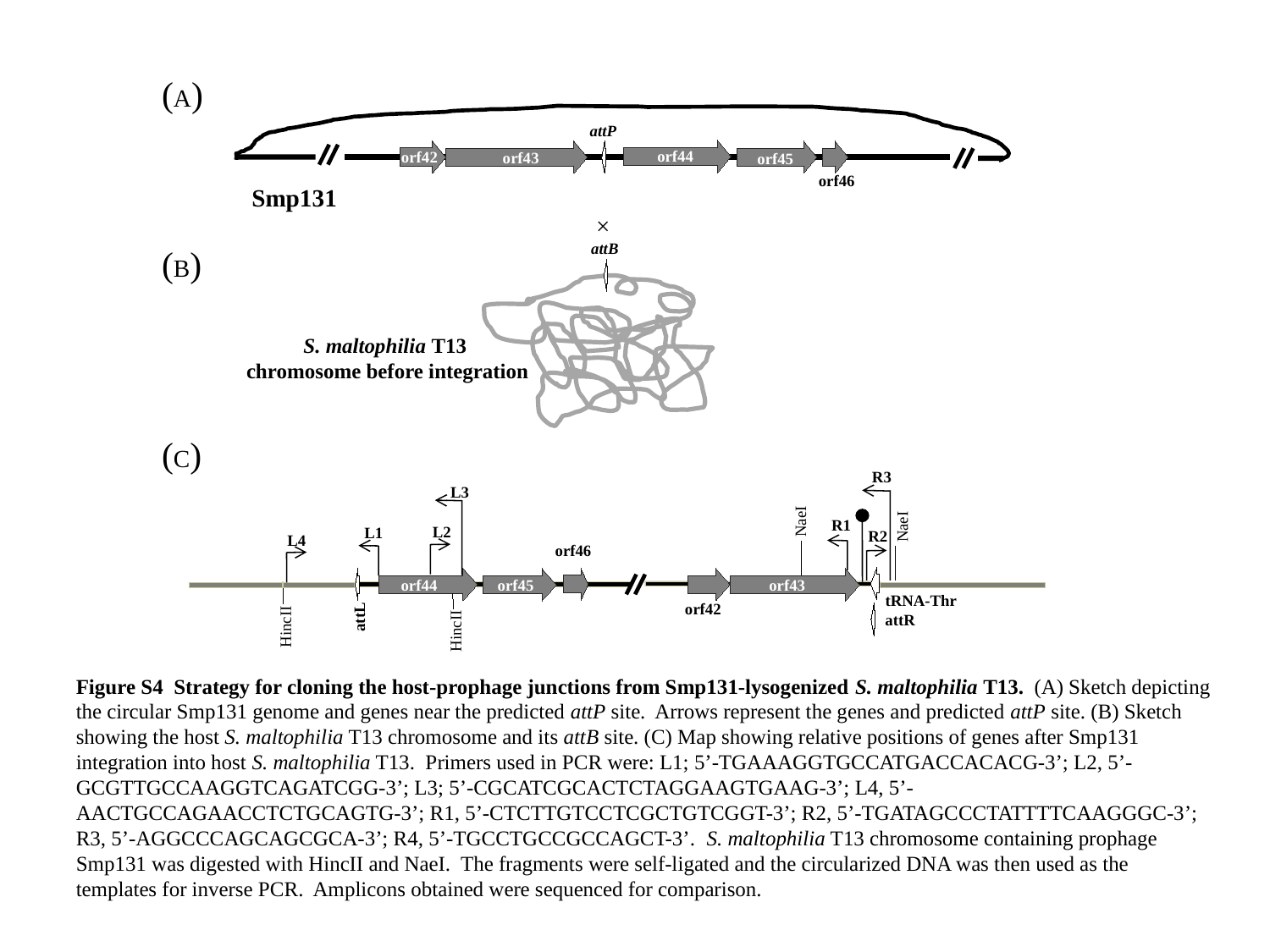

(A)
attP
orf44
orf42
orf43
orf45
orf46
×
Smp131
(B)
attB
S. maltophilia T13
chromosome before integration
(C)
R3
L3
NaeI
NaeI
R1
L2
L1
L4
R2
orf46
orf44
orf43
orf45
tRNA-Thr
orf42
attL
attR
HincII
HincII
Figure S4 Strategy for cloning the host-prophage junctions from Smp131-lysogenized S. maltophilia T13. (A) Sketch depicting the circular Smp131 genome and genes near the predicted attP site. Arrows represent the genes and predicted attP site. (B) Sketch showing the host S. maltophilia T13 chromosome and its attB site. (C) Map showing relative positions of genes after Smp131 integration into host S. maltophilia T13. Primers used in PCR were: L1; 5’-TGAAAGGTGCCATGACCACACG-3’; L2, 5’-GCGTTGCCAAGGTCAGATCGG-3’; L3; 5’-CGCATCGCACTCTAGGAAGTGAAG-3’; L4, 5’-AACTGCCAGAACCTCTGCAGTG-3’; R1, 5’-CTCTTGTCCTCGCTGTCGGT-3’; R2, 5’-TGATAGCCCTATTTTCAAGGGC-3’; R3, 5’-AGGCCCAGCAGCGCA-3’; R4, 5’-TGCCTGCCGCCAGCT-3’. S. maltophilia T13 chromosome containing prophage Smp131 was digested with HincII and NaeI. The fragments were self-ligated and the circularized DNA was then used as the templates for inverse PCR. Amplicons obtained were sequenced for comparison.
